# Two Obstetrical Cases in Dwarfs

**Published:** 1859-10

**Authors:** A. J. Shaffer

**Affiliations:** Lawrenceville, Georgia


ATLANTA
Medical and Surgical Journal.

VOL. V.] OCTOBER, 1859. [NO. 2.

### ORIGINAL COMMUNICATIONS.

### ARTICLE I.

*Two Obstetrical Cases in Dwarfs.* By A. J. SHAFFER, M. D., of Lawrenceville, Georgia.

I purpose at this time to give you a short history of two cases of Obstetrics, that happened in my practice, thinking they will not be without interest to the profession, and your readers generally. The patients being dwarfs, and being well known throughout the State of Georgia, and several of the adjoining States. It is not a little strange that the father of those patients is the first dwarf that occurred in either family of his ancestry, (to the best of his knowledge,) and that one half of his family were dwarfs. He is in statue three and a half feet, heavy body, short arms and legs, and a more than ordinary large head; his average weight one hundred pounds; he married a lady of little over medium height, and spare made. They have had six children, five daughters and one son—three of the daughters were dwarfs, and two of the average size; the two latter are both married, and raising families of stout, well formed children. The son died at the age of five years, and was a large, well formed child. The second of the dwarfs married at the age of seventeen, moved to the State of Alabama, where she died in child-bed, undelivered; she was in heighth fortythree inches. Emeline, the eldest, married in 1857, in the State of Tennessee, became pregnant; three months before her confinement, she returned to her parents, who live eight miles from this place, for the purpose, as they said, to have the advantage of Dr. Russell's professional services in the time of her confinement.

August 1st, 1858.—Dr. Russell was called to see her at 10, p. m., and found her having premonitory symptoms of labor, which she stated she had been experiencing for some four or five hours; the os was slightly dilated; the case not being urgent he gave her a dose of oil and retired—after sending for me.

2d, 6, a. m.—Labor had fairly set in; the little patient was aged 28, plethoric habit, height thirty-eight inches, arms and legs very short, small bones, usual weight seventy pounds. On examination per vaginum, (digital,) I found the following phenomena existing: External organs of generation well developed; the vaginal canal very small, in consequence of the abnormal formation of the sacrum and coxcix, they being quite straight; hence the concavity was hardly perceptible; small pelvis, the prominence of the sacrum and os pubis approximated each other so closely that the antero-posterior diameter was, according to the best of our judgment, one and one fourth inches; and the transverse diameter two and one half inches; the os uteri well dilated and flexible; the vertex presented, cranial bones well ossified, so much so that the fontanels were hardly perceptible. I proposed to Dr. R. the cæsarean section, the surest operation to save the life of the child, and possibly that of the mother; but he was of the opinion he could dissect the child away, but in either case it was necessary to have more counsel and assistance. We sent for Drs. Moore and McBeen—on general consultation, they agreed with Dr. Russel, to attempt the delivery by dissection. (It is proper for me to state that I dissented from that decision, for obvious reasons.)

At 12, a. m., Dr. Russel perforated the cranium, which caused little pain and a moderate flow of blood. With great difficulty he succeeded in removing the parietals, part of the os frontis and temporals. After an effort of three hours dissection was abandoned as hopeless, the bones being too completely ossified, and the outlet too contracted to use any instruments at our command, successfully. The patient bore the attempt at dissection remarkably well, without the use of chloroform. There was but one operation that afforded the little patient and her friends a hope for her relief, that was cæsarian section, which she readily submitted to. She was placed on a table, and being put fully under the influence of chloroform, I made an incision in the *linea-alba*, in length four inches, the peritoneum being reached, the hemorrhage which was quite trifling, not more than half an ounce, being stopped, the peritoneum was divided, the womb being flexible, I made an incision in that organ, corresponding in length with that in the parietes, the womb remaining flexible, I seized the feet of the fœtus and dislodged it, but not without some difficulty, from the necessary smallness of the wounds, and a large fœtus, which, if living, would have weighed nine pounds. The placenta was detached almost spontaneous with the removal of the fœtus, by the very active contraction of the uterus, which caused considerable hemorrhage, but was prevented from passing into the peritoneal cavity, by the edges of the abdominal wound being pressed down tightly around that in the womb, and by the free use of the sponge. The contraction of the womb was active, strong and regular, so that in fifteen minutes the wound was reduced to one and a half inches in length, and not gapping more than one fourth of an inch. A coagula being formed around the internal edge, which effectually prevented the escape of blood into the peritoneal cavity, by a proper care to keep the patient on her back for some hours. We proceeded to dress the abdominal wound, the patient at this time not fully under the influence of chloroform, became nauseated; made several violent attempts to vomit, which, notwithstanding our efforts to prevent it, several loops of the bowels were forced through the wound, which proved quite troublesome in dressing the wound, which was done by applying six sutures, four strips of adhesive plaster, a compress of domestic cloth, and a wide bandage drawn moderately tight around the abdomen, and the patient placed comfortably in bed. She was under the influence of chloroform one hour, when she awoke, said she felt more comfortable than she had expected, but soon became quite weak; gave her chicken broth, with a little brandy, which had a fine effect. At 10, p. m., became quite restless—gave her sulphate morphia, gr. $\frac{1}{4}$; dover's powder, gr. iv, which gave her three hours sleep.

3d, 8, a. m., commenced swelling this morning—gave her an enema spts. turpentine, ℥iss.; gum musilage, Oi., which produced two evacuations. Calomel, gr. x.; dov. powder, gr. xiv.; sulphate morphine, gr. i, M., divided into four powders, one to be taken every four hours.

4th, 6, a. m., slept but little last night, pulse 130, expression of countenance anxious, difficult breathing, the lachial discharge stopped, the superior part of the wound adhered about one inch, by first intention, the lower part quite unhealthy, the abdomen continues to swell and is quite sensitive to the touch, no operation since the enema, gave her castor oil, ℥i.; spts, turpentine, ʒi., which operated twice; operations dark. 8, p. m., the patient sinking, pulse 115. 5th, died $3\frac{1}{2}$ a. m., $63\frac{1}{2}$ hours after the operation.

Case 2d.—*Sept. 8th*, 1858.—Dr. Russell was called to see Miss Amanda Janes, sister of the above case. She complained of having pain in the left aliac and hypochondrial region. In getting a history of the case, her mother represented her as having taken cold about eight months since; that she had not menstruated since that time. After an examination, he informed the parents that the patient was pregnant, and was of the opinion that the pains she was then having were symptomatic of labor; that he was strongly of opinion that she had gone her full time.

Notwithstanding all this, the patient contended that she was not pregnant; that her complaint was nothing but cold she had contracted at the time above stated. He gave her an opiate and left several powders for her to take at any time that she might have pain. Dr. R. left, telling them to send for him the next day if she did not get better, and with the understanding that we would call to see her in three or four days.

On the 8th, I accompanied Dr. Russell; found the patient sitting up; complained of some pain in her back, and, as she said, in the lower part of her bowels. Age 18 years, heighth 41 inches, of plethoric habit, usual weight 80 lbs. On digital examination, pr. vaginam, the same abnormal condition of the parts presented, as in the first case, the anterio-posterior diameter of the pelvic outlet one and a half inches; transverse, two and a half inches; the os undilated; the movements of the fetus was strong; and all the signs were indicative of eight months in utero. As there was no chance for her to give birth to a living child, we determined to produce premature labor by rupturing the membrane, which was done by introducing a bougie, directed her a light, nutritious diet, to keep her bowels regular with oil, and to send for us when labor or bearing down pains set in.

Sept. 12th, 6 A. M., I was sent for in great haste. On my arrival, I found the patient cheerful; said she had been having pains since 12 o'clock last night. The os was slightly dilated. At 11, A. M., bearing down pains set in; at 4, P. M., the os had dilated to the size of a quarter of a dollar; at 10, P. M., labor having fairly set in, the os well dilated and flexible, I sent for assistance. My friend Dr. Russell, having gone to Hall county, I sent back for Drs. K. Mitchell and R. Knox; gave the patient morphine, gr. ss., which relieved her of the constant pain, and gave her a full hours sleep.

13th, 7 A. M.—Drs. Mitchell and Knox arrived. The patient being quite weak at this time, we gave her some wine and chicken broth. 8½, she was put fully under the influence of chloroform by Dr. Mitchell. After perforating the cranium, I commenced the dissection, by the use of the crotchet, or short hook, and by taking hold between the sutures, each bone (craniel) was removed in regular order, commencing with the paretal; with the same instrument I drew down the arms, and used them for instruments of traction. I succeeded in dislodging the fetus, which, if living, would have weighed 8½ lbs., and was at least eight and a half months in utero. The placenta was detached by the active contractions of the womb. The patient was under the influence of chloroform one and a half hours. The hemorrhage, as might be expected, was moderate. She was placed in bed, and expressed great satisfaction in feeling so well, and a hope of getting well.

14th.—Rested tolerably well last night, after taking morphine; has some pain in the left aliac region; has a good deal of tenderness on pressure, and some swelling of the abdomen; frequent disposition to mixturate, scanty and high collera, pulse 100.

*Prescription*—Calomel, gr. x.; Dover's powders, gr. xvi.; morphia sulphate, gr. i.; mix, and divide into four powders—one to be taken every four hours; Bals. cop., ℥i.; Spts. niter, ℥i.; mix—25 drops to be taken every six hours. Emolient poultice applied over the hypogastrum.

15th.—Rested quite well last night; generally better; has less tenderness of the abdomen; miturates less frequent and with more ease, but frequent watery discharges from the bowels; pulse 95 per minute. Prescription continued.

16th.—Bowels less aetive and more bilious; very little swelling of the abdomen; has a cutting pain in the left aliac region; pulse 95 per minute. Applied a large blister over the seat of pain; cast. oil, ℥ ss.; spts. turpentine, ℥i.; morphine, gr. i.; Dover's powder, xii.; mix and divide into four powders, one to be taken every six hours. After the oil operates, take four gr. of blue pill to-night.

17th.—The blister had the desired effect; has had no pain since it drew; bowels quiet; pulse 90 per minute. Powders and bals. continued.

19th.—Has no bad symptoms since my last visit. Appetite improving; has some soreness on pressure over the womb; pulse 88 per minute. Dov. powders, gr. xxiv.; sulph. morphia, gr. ii.; mix, and divide into six powders, one to be taken each night, or at any time that she might have pain or feel restless. Cals. mixture, 15 drops three times per day.

23d.—Taken all the powders. This treatment was continued up to the 30th, at which time I dismissed the patient convalescing, which was a happy one, and she has since married.

P. S.—I have no doubt in my own mind that if we had have performed the Cæsarean section before any attempt at dissection in the first case, the patient would have recovered, for I am confident that the perotoneal inflammation was produced by the attempt at dissection. It is not necessary for me to say why dissection failed, if it is borne in mind the smallness of the outlet, and thorough ossification of the bones of the cranium, so much so that the fortunels were almost obliterated. The patient was satisfied that she carried it ten months. It must farther be borne in mind that puerperal peritonitis was quite prevalent in that neighborhood at the time, of which a good many women died.

